# Lymphœdème unilatéral du membre supérieur au cours d'une polyarthrite rhumatoïde

**DOI:** 10.11604/pamj.2015.21.214.2708

**Published:** 2015-07-24

**Authors:** Salem Bouomrani, Hanène Nouma, Alaeddine Slama, Safouane Chebbi, Marwa Neffoussi, Afef Fara, Maher Beji

**Affiliations:** 1 Service de Médecine Interne, Hôpital Militaire de Gabès, 6000 Gabès, Tunisie

**Keywords:** Polyarthrite rhumatoïde, lymphœdème chronique, lymphographie, lympho-scintigraphie, Rheumatoid arthritis, chronic lymphedema, lymphography, lympho-scintigraphy

## Abstract

Les lymphœdèmes chroniques et localisés des membres ne sont qu'exceptionnellement signalés au cours de la polyarthrite rhumatoïde (PR). Nous rapportons l'observation d'une patiente âgée de 63 ans ayant une PR diagnostiquée depuis dix ans et traitée par hydroxychloroquine, prednisone et méthotrexate avec une bonne évolution fût explorée pour une tuméfaction du membre supérieur gauche évoluant depuis deux ans. À l'examen clinique on notait un membre infiltré en totalité, indolore, élastique et recouvert d'une peau tendue, luisante mais d'aspect normal. Le reste de l'examen somatique était sans particularités. La biologie ne montrait pas d'anomalies. L'imagerie médicale (radiographies-X standards des os de l'avant bras et du thorax, scanner-X thoracique, échographie des parties molles et du creux axillaire, doppler artériel et veineux du membre atteint et écho-mammographie) se révélait normale. La lympho-scintigraphie concluait à l'absence de visualisation du réseau lymphatique superficiel gauche. Le diagnostic de lymphœdème secondaire associé à la PR était retenu devant la négativité du bilan étiologique. Une kinésithérapie de drainage lymphatique fût prescrite en association à des assauts cortisoniques mais l'amélioration n’était que partielle. Parmi les manifestations extra articulaires de la PR, les lymphœdèmes chroniques localisés des membres restent inhabituels et souvent méconnus. Leurs mécanismes physiopathologiques sont mal élucidés et leur traitement ne fait pas encore l'unanimité. Ils gardent en revanche une implication pronostique fonctionnelle majeure.

## Introduction

Les lymphœdèmes chroniques secondaires des extrémités sont extrêmement rares au cours de la PR [1–3] et sont d'individualisation relativement récente: la première observation de lymphœdème au cours de la PR fût rapportée par Kalliomaki et Vastamaki en 1968 [4]. Depuis seulement quelques cas isolés sont rapportés et la PR est actuellement reconnu parmi les étiologies rares des lymphœdèmes secondaires localisés [5]. Ces lymphœdèmes sont par ailleurs signalés dans d'autres rhumatismes inflammatoires chroniques voisins de la PR; en particulier le rhumatisme psoriasique [6–8] et l'arthrite chronique juvénile [9, 10]. Une observation originale de lymphœdème localisé de membre fût rapportée chez une patiente ayant une PR associée à un rhumatisme psoriasique [8] renforçant une fois de plus le rôle présumé de ces rhumatismes inflammatoires chroniques dans la genèse d'un tel désordre. A travers notre observation nous discutons les différents aspects pathogéniques, cliniques et thérapeutiques de cette association inhabituelle.

## Patient et observation

**Figure 1:**
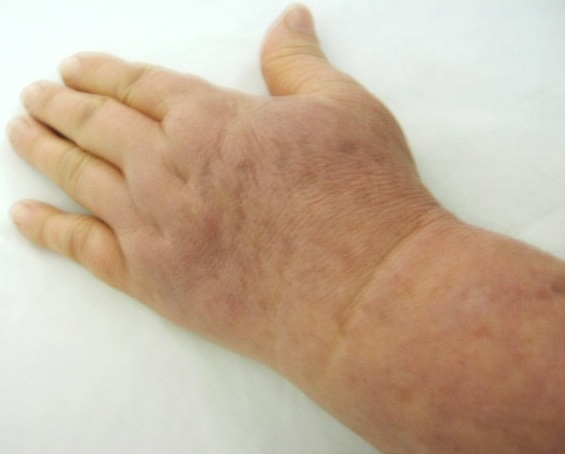
Lymphœdème prenant la totalité de la main.

**Figure 2:**
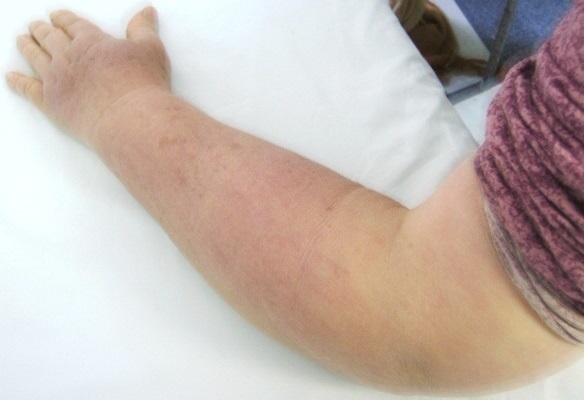
Lymphœdème de l'avant bras remontant jusqu'au mi bras gauche.

Patiente S.F. âgée de 63 ans ayant une PR diagnostiquée depuis dix ans devant une polyarthrite inflammatoire des grosses et petites articulations, une carpite fusionnante bilatérale radiologique et un facteur rhumatoïde fortement positif, et traitée par hydroxychloroquine 200 mg/j, prednisone 10 mg/j et méthotrexate 10 mg/semaine avec une bonne évolution clinique et biologique fût explorée pour une tuméfaction du membre supérieur gauche (MSG) évoluant depuis deux ans. À l'examen clinique on notait un membre infiltré en totalité, indolore, élastique et recouvert d'une peau tendue, luisante mais d'aspect normal (Figure 1 et Figure 2). Le reste de l'examen somatique était sans anomalies, en particulier pas de syndrome tumoral, pas d'adénopathies axillaires ni de cordons veineux palpables. La biologie s'est révélée normale, en particulier pas de syndrome inflammatoire (vitesse de sédimentation, protéine C réactive et électrophorèse des protéines sériques), pas d'anomalies de la numération formule sanguine, enzymes musculaires à des taux normaux et bilan immunologique négatif (anticorps anti nucléaires, complexes immun circulants, fractions C2, C3, C4, CH50 et C1-inhibitor du complément). Les marqueurs tumoraux étaient négatifs (CA125, CA15-3, ACE et aFP). Les radiographies standards des os de l'avant bras et du thorax étaient sans lésions. Le scanner X thoracique était normal ainsi que l’échographie des parties molles et du creux axillaire. Le doppler veineux du membre n'a pas montré de signes d'insuffisance veineuse ni de thrombose profonde ou superficielle concomitante. Le doppler artériel n'a pas montré non plus de signes d'artériopathie sous-jacente. L’écho-mammographie s'est révélé normale. La lympho-scintigraphie au nano-colloïde marqué concluait à l'absence de visualisation du réseau lymphatique superficiel gauche. Le diagnostic de lymphœdème localisé du MSG associé à la PR était retenu devant la négativité du bilan étiologique. Une kinésithérapie de drainage lymphatique fût prescrite puis un mini bolus de méthylprednisolone (500 mg/j pendant trois jours de suite) devant la non amélioration par la kinésithérapie. L'amélioration n’était que partielle avec persistance de l’œdème et de la gène fonctionnelle.

## Discussion

Les lymphœdèmes secondaires localisés et chroniques des membres sont actuellement considérés par certains auteurs comme une manifestation extra articulaire originale et inhabituelle de la PR [11, 12]. Ils touchent aussi bien les membres inférieurs que supérieurs avec une nette prédominance pour ces derniers [2, 12, 13]; l'atteinte des jambes reste exceptionnelle [12, 14, 15] et les formes bilatérales sont possibles mais rares [2, 12, 16].

Ces lymphœdèmes surviennent classiquement au cours de l’évolution de la PR [12] mais des formes inaugurales de la maladie ont été rapportées [17].

Il n'a pas été observé de corrélations avec le taux du facteur rhumatoïde ni l'activité clinique de la maladie [1, 12, 18], ni sa durée d’évolutivité [18]. Parfois l'apparition de ces lymphœdèmes est concomitante aux poussées de la maladie rhumatismale [11]. La pathogénie de cette manifestation demeure obscure [2]. La théorie la plus évoquée est celle de l'obstruction des canaux lymphatiques causée par la fibrine et les produits de dégradation des autres facteurs physiologiques de la coagulation [1]. Cette hypothèse trouve ces bases dans la constatation, chez les rhumatoïdes ayant des lymphœdèmes, d'une élévation nette des concentrations plasmatiques des produits de dégradation du fibrinogène (PDF): 4/6 des patients de Minari C. et al. [1]. Par ailleurs d'autres mécanismes physiopathogéniques ont été évoqués: la destruction-obstruction des canaux lymphatiques par l'inflammation persistance via une lymphangite chronique [2, 12]; l'augmentation de la perméabilité lymphatique locale [12]: cette hypothèse trouve ses fondements dans les anomalies de la perméabilité capillaire déjà prouvées au cours de la PR avec augmentation du coefficient de filtration capillaire [19]. De même l'association à d'autres pathologies et/ou conditions qui augmentent la perméabilité capillaire (diabète et para protéine de type IgM) chez les rhumatoïdes présentant des lymphœdèmes secondaires chroniques renforce cette hypothèse [12]; l'implication directe de la synovite locale dans la genèse de ces lymphœdèmes a aussi été évoquée au cours des rhumatismes inflammatoires chroniques; en occurrence le rhumatisme psoriasique [6]. Mais souvent le lymphœdème est très étendu débordant largement l'aire de l'articulation sous jacente et rendant ainsi la synovite locale insuffisante à elle seule pour expliquer son apparition [20] et laissant évoquer d'autres facteurs favorisants. Cette synovite agit via une lymphangite oblitérante qui entrave le drainage des lymphatiques synoviaux [21]; par ailleurs le caractère localisé de ces lymphœdèmes permet de ne pas les rattacher à certains facteurs généraux favorisant l'infiltration œdémateuse et qui sont souvent retrouvés au cours de la PR: hypoalbuminémie, anémie et rétention hydrosodée [20]; enfin un cas anecdotique de lymphœdème survenant chez un patient présentant une polyarthrite inflammatoire active associée à une hypoplasie lymphatique congénitale a été rapporté laissant penser à un déterminisme héréditaire avec une éventuelle prédisposition génétique pour une telle association [22]?

Le diagnostic de ces lymphœdèmes est principalement clinique basé sur l'aspect infiltré, tuméfié, peu douloureux et tendu du membre atteint avec une lourdeur et une impotence fonctionnelle variable [2]. La confirmation sera faite par la biopsie de nœuds lymphatiques du territoire de drainage, la lymphographie ou la lympho-scintigraphie; cette dernière reste la plus pratiquée et la plus rentable [2]. L'imagerie révèle classiquement une obstruction des canaux lymphatiques profonds [1, 13, 20]; plus rarement des ectasies lymphatiques peuvent être notées [11]. La régression qu'elle soit spontanée ou sous traitement est rarement observée et la chronicité est souvent de règle [12]. Ces lymphœdèmes vont ainsi faire le lit à plusieurs complications sévères et parfois invalidantes; en particulier les surinfections bactériennes, les limitations articulaires et principalement les ulcères chroniques de peau [23, 24]. En effet 11 patients sur 36 dans la série de Seitz CS et al. d'ulcères chroniques de jambes au cours de la PR avaient un lymphœdème chronique secondaire sous jacent [23].

La prise en charge thérapeutique de ces lymphœdèmes est trop controversée et est souvent décevante [2]. Il est connue par ailleurs que le traitement de la PR sous jacente ne les améliore pas [2]. Le traitement physique parait être le plus efficace [25] et l'efficacité du drainage lymphatique peut être amélioré par l'adjonction d'un diurétique [1]. Les injections locales de corticoïdes dans les articulations juxta lymphatiques ont été aussi essayées avec succès permettant dans certains cas la résolution complète de ces lymphœdèmes [8]. Ces résultats renforcent encore une fois l'hypothèse étiologique impliquant la synovite locale dans la genèse des lymphœdèmes au cours des rhumatismes inflammatoires chroniques [6]. La chirurgie fût anciennement proposée comme moyen thérapeutique avec des résultats satisfaisants dans 50% des cas [14].

Actuellement la biothérapie; principalement les anti TNFa, occupent une place de choix dans le traitement de ces lymphœdèmes associés aux rhumatismes inflammatoires chroniques avec des résultats très satisfaisants [15, 26–29]; surtout si résistance à la corticothérapie systémique. Ceci dit il faut garder à l'esprit la possibilité de voir induire des lymphœdèmes secondaires chez les patients rhumatoïdes traités par les anti TNFa; c'est l'une des réactions dites « paradoxales » des anti TNF: jusqu’à nos jours un seul cas de lymphœdème induit par l'Etanercept^®^ a été rapporté au cours de la PR [30].

## Conclusion

Le lymphœdème secondaire localisé et chronique de membres reste une manifestation extra articulaire exceptionnelle de la polyarthrite rhumatoïde. Souvent méconnu et négligé, il représente un facteur pronostique majeur, surtout fonctionnel et particulièrement dans les formes surinfectées ou compliquées d'ulcères chroniques de jambes. La prise en charge est souvent lourde, prolongée et multidisciplinaire au sein de laquelle le drainage lymphatique, la corticothérapie et les anticorps monoclonaux occupent la place de choix.

## References

[1] Minari C, Cecconami L, Fioravanti A, Montemerani M, Scola C, Marcolongo R (1994). Lymphoedema of the limbs in rheumatoid arthritis. Clin Rheumatol..

[2] Joos E, Bourgeois P, Famaey JP (1993). Lymphatic disorders in rheumatoid arthritis. Semin Arthritis Rheum..

[3] Lannes F, Sailler L, Ollier S, Carreiro M, Arlet P (2001). Lymphedema of the upper limb, a complication of rheumatoid polyarthritis. Presse Med..

[4] Kalliomäki JL, Vastamäki M (1968). Chronic diffuse oedema of the rheumatoid hand: a sign of local lymphatic involvement. Ann Rheum Dis..

[5] Vignes S (2010). Secondary limb lymphedema. Presse Med..

[6] Mulherin DM, FitzGerald O, Bresnihan B (1993). Lymphedema of the upper limb in patients with psoriatic arthritis. Semin Arthritis Rheum..

[7] Böhm M, Riemann B, Luger TA, Bonsmann G (2000). Bilateral upper limb lymphoedema associated with psoriatic arthritis: a case report and review of the literature. Br J Dermatol..

[8] Bamji A (1991). Limb lymphoedema in rheumatoid arthritis. Ann Rheum Dis..

[9] Ambrósio C, Abreu P, Alexandre M, Malcata A (2008). Lymphoedema in systemic juvenile arthritis: a rare extraarticular feature. Acta Reumatol Port..

[10] Schmit P, Prieur AM, Brunelle F (1999). Juvenile rheumatoid arthritis and lymphoedema: lymphangiographic aspects. Pediatr Radiol..

[11] Hidalgo Calleja C, Cuesta Andrés M, Llorente Melero MJ, Espinosa Castelló A, Balsa Criado A, Gijón Baños J (1993). Lymphoscintigraphic study in a case of rheumatoid arthritis-related lymphoedema. Clin Exp Rheumatol..

[12] Dacre JE, Scott DL, Huskisson EC (1990). Lymphoedema of the limbs as an extra-articular feature of rheumatoid arthritis. Ann Rheum Dis..

[13] Sant SM, Tormey VJ, Freyne P, Casey EB (1995). Lymphatic obstruction in rheumatoid arthritis. Clin Rheumatol..

[14] Ignjativic N, Cerovic S (1999). Lymphedema of the leg associated with rheumatoid arthritis. Vojnosanit Pregl..

[15] Eyigor S, Karapolat H, Kirazli Y (2008). Efficacy of etanercept and complete decongestive physical therapy in bilateral lower-limb lymphoedema associated with rheumatoid arthritis: a case report. Adv Ther..

[16] Kyle VM, De Silva M, Hurst G (1982). Rheumatoid lymphoedema. Clin Rheumatol..

[17] Nagai Y, Aoyama K, Endo Y, Ishikawa O (2007). Lymphedema of the extremities developed as the initial manifestation of rheumatoid arthritis. Eur J Dermatol..

[18] Kiely PD, Joseph AE, Mortimer PS, Bourke BE (1994). Upper limb lymphedema associated with polyarthritis of rheumatoid type. J Rheumatol..

[19] Jayson MI, Barks JS (1971). Oedema in rheumatoid arthritis: changes in the coefficient of capillary filtration. Br Med J..

[20] De Silva RT, Grennan DM, Palmer DG (1980). Lymphatic obstruction in rheumatoid arthritis: a cause for upper limb oedema. Ann Rheum Dis..

[21] Kuhns JG (1933). Lymphatic drainage of joints. Arch Surg..

[22] McRorie ER, Reid DM, Dunn NA, Nuki G (1995). Lymphoedema associated with active inflammatory arthritis in a patient with congenital lymphatic hypoplasia. Br J Rheumatol..

[23] Seitz CS, Berens N, Bröcker EB, Trautmann A (2010). Leg ulceration in rheumatoid arthritis--an underreported multicausal complication with considerable morbidity: analysis of thirty-six patients and review of the literature. Dermatology..

[24] Vaillant L, Müller C, Goussé P (2010). Treatment of limbs lymphedema. Presse Med..

[25] Kogure T, Hoshino A, Ito K, Sato H, Tatsumi T, Ohyama Y, Kawata E, Fujita K, Tamura J (2005). Beneficial effect of complementary alternative medicine on lymphedema with rheumatoid arthritis. Mod Rheumatol..

[26] Tong D, Eather S, Manolios N (2009). Psoriatic arthritis and chronic lymphoedema: treatment efficacy by adalimumab. Clin Rheumatol..

[27] Lekpa FK, Economu-Dubosc A, Fevre C, Claudepierre P, Chevalier X (2009). Efficacy of etanercept in lymphedema associated with psoriatic arthritis. J Rheumatol..

[28] Ostrov BE (2001). Beneficial effect of etanercept on rheumatoid lymphedema. Arthritis Rheum..

[29] Doffoël-Hantz V, Sparsa A, Verbeke S, Tricard MJ, Bonnetblanc JM, Bédane C (2007). Lymphoedema: a rare complication of inflammatory rheumatism. Successful treatment with etanercept. Eur J Dermatol..

[30] Tily HI, Perl A (2009). Lymphoedema: a paradoxical effect of tumour necrosis factor inhibitors - case report and review of literature. BMJ Case Rep..

